# Hierarchical Microbial Functions Prediction by Graph Aggregated Embedding

**DOI:** 10.3389/fgene.2020.608512

**Published:** 2021-01-18

**Authors:** Yujie Hou, Xiong Zhang, Qinyan Zhou, Wenxing Hong, Ying Wang

**Affiliations:** ^1^Department of Automation, Xiamen University, Xiamen, China; ^2^Department of Automation, University of Science and Technology of China, Hefei, China; ^3^School of Automation Science and Engineering, South China University of Technology, Guangzhou, China; ^4^Institute of AI and Robotics, Fudan University, Shanghai, China; ^5^Xiamen Key Laboratory of Big Data Intelligent Analysis and Decision, Xiamen, China; ^6^Fujian Key Laboratory of Genetics and Breeding of Marine Organisms, Xiamen, China

**Keywords:** microbial co-occurrence networks, functions prediction, graph embedding, hierarchical multi task learning, deep learning

## Abstract

Matching 16S rRNA gene sequencing data to a metabolic reference database is a meaningful way to predict the metabolic function of bacteria and archaea, bringing greater insight to the working of the microbial community. However, some operational taxonomy units (OTUs) cannot be functionally profiled, especially for microbial communities from non-human samples cultured in defective media. Therefore, we herein report the development of Hierarchical micrObial functions Prediction by graph aggregated Embedding (HOPE), which utilizes co-occurring patterns and nucleotide sequences to predict microbial functions. HOPE integrates topological structures of microbial co-occurrence networks with *k*-mer compositions of OTU sequences and embeds them into a lower-dimensional continuous latent space, while maximally preserving topological relationships among OTUs. The high imbalance among KEGG Orthology (KO) functions of microbes is recognized in our framework that usually yields poor performance. A hierarchical multitask learning module is used in HOPE to alleviate the challenge brought by the long-tailed distribution among classes. To test the performance of HOPE, we compare it with HOPE-one, HOPE-seq, and GraphSAGE, respectively, in three microbial metagenomic 16s rRNA sequencing datasets, including abalone gut, human gut, and gut of *Penaeus monodon*. Experiments demonstrate that HOPE outperforms baselines on almost all indexes in all experiments. Furthermore, HOPE reveals significant generalization ability. HOPE's basic idea is suitable for other related scenarios, such as the prediction of gene function based on gene co-expression networks. The source code of HOPE is freely available at https://github.com/adrift00/HOPE.

## Introduction

The analysis of microbial communities is founded on the characterization of functional diversity, which is increasingly recognized as the bridge linking biodiversity patterns and ecosystem functioning, as a way of explaining the interactions between microbes and their responses to changes in the environment (Bardgett and Der Putten, 2014; Escalas et al., 2019). However, a large proportion of microbes remain uncultivated and, therefore, functionally unknown. However, because of the prevalence of high-throughput sequencing technologies, large-scale 16S rRNA marker gene sequencing of microbes is becoming available. Related approaches, such as PICRUSt (Langille et al., 2013) and Tax4Fun (Ashauer et al., 2015), are proposed to infer functional profiles from genomes and phylogeny. PICRUSt and Tax4Fun identify microbial functions by estimating 16s rRNA marker gene families based on the similarity between 16s rRNA sequencing data and known marker gene databases. They rely on the reference databases on Greengenes (Desantis et al., 2006) and SILVA (Quast et al., 2012). However, owing to the incompleteness of the 16S rRNA marker gene database, large amounts of OTUs cannot be functionally profiled, especially for microbial communities from non-human samples from defective culture media (Pachiadaki et al., 2019; Wang X. et al., 2020).

The protein–protein interaction (PPI) network in protein function prediction gives theoretical insight to our study of microbial functional diversity. More specifically, network representation of the PPI network extracts functional context from topological structure (Gligorijevic et al., 2018; Kulmanov et al., 2018) and achieves better performance than the previous algorithm that only uses sequence data (Wass et al., 2012; Cozzetto et al., 2013). Several researchers found that proteins with interactions in PPI networks have a high possibility of sharing the same or similar functions (Lele et al., 2011; Liu et al., 2017). Inspired by empirical success in using the PPI network, we can build a microbial co-occurrence network to provide new insights into the exploration of microbial functions. Microorganisms do not live in isolation but, rather, interact with the environment through, for example, mutualism, competition, parasitism, and predation. “Co-occurrence” means that microbes have statistically significant associations of abundance in one microbial community. The co-occurrence relationship is generally inferred by abundance correlation over several microbial community samples. The microbial co-occurrence network was designed to describe these relationships among microbes, and those microbes with closely correlated relationships become linked in the microbial co-occurrence network.

A novel method, Hierarchical micrObial functions Prediction by graph aggregated Embedding (HOPE), was proposed to capture potential functions in a microbial co-occurrence network. Our method is built based on the key hypothesis that microbes with co-occurring patterns have a high possibility of sharing the same or similar functions. So, our method tries to use this property to infer unknown microbe functions from its neighbors in the microbial co-occurrence network. HOPE has two main modules: hierarchical multitask learning and graph embedding. Here, the hierarchical multitask learning framework solves the class imbalance problem, and the graph embedding learns the co-occurrence patterns in microbial networks. Two classic strategies have traditionally been performed: resampling (Chawla et al., 2002) and cost-sensitive reweighting (Khan et al., 2018) during our previous experiments. These methods change the training dataset distribution by either undersampling the majority class, oversampling the minority class, or giving a higher cost to misclassification of the minority class. However, neither of these classic methods could ameliorate the negative impact of imbalanced classes during our experiments. Both the majority class and the minority class can be well-classified if they are trained independently; therefore, we were motivated to design a hierarchical multitask training scheme to manage the imbalance of functional datasets with the long-tailed distribution. To accomplish this, we input two graphs with the majority class and the minority class, respectively, into the HOPE algorithm and train the model by multitask learning. A graph embedding model is designed to map the microbial co-occurrence network to a lower-dimensional continuous latent space while maximally preserving the topological relationships among OTU features. HOPE incorporates *k*-mer compositions of microbial sequences and topology of microbial networks, as complementary data sources, to learn an embedding representation of a microbial network. The embedded low-dimensional numerical vector of each OTU node reflects its sequencing features and co-occurrence correlation with its neighbors. After that, the multilayer perceptron (MLP) classifier takes embedding vectors as inputs to predict the function for those OTUs without functional information from the known database.

Cross-validation was designed to evaluate the performance of HOPE on three microbial metagenomic 16s rRNA sequencing datasets from abalone gut, human gut, and gut of *Penaeus monodon*, respectively, and all experiments mentioned above verified the superiority of HOPE in predicting microbial functions. HOPE is compared with its two variants, HOPE-seq and HOPE-one, as well as a well-known graph embedding algorithm, GraphSAGE (Hamilton et al., 2017), in the experiments. HOPE-seq uses only *k*-mer frequency vectors as features with the hierarchical multitask learning framework to train the classifier on majority classes and minority classes. HOPE-one ignores the hierarchical multitask learning but integrates the sequence representation with microbial network topological structure as embedding features for function prediction. In the testing set, we learned that HOPE outperforms HOPE-seq and HOPE-one on almost every measurement. HOPE outperforms HOPE-one by 9.5% in Micro-F1 on the Abalone Gut Microbiota dataset and 15.6% in Macro-F1 on the *P. monodon* intestine dataset. When compared with GraphSAGE, using three different aggregator functions, including a mean aggregator, an LSTM aggregator, and a pooling aggregator, HOPE achieves the highest score in most measurements with a significant margin. Compared with GraphSAGE, HOPE gains a higher accuracy score by 4.4% averagely. Finally, our results show that HOPE demonstrates significant generalization ability since it can be used to predict microbial functions without learning previous information in our experiments.

## Methods

### Framework of Microbial Function Prediction With HOPE

**Figure 1:**
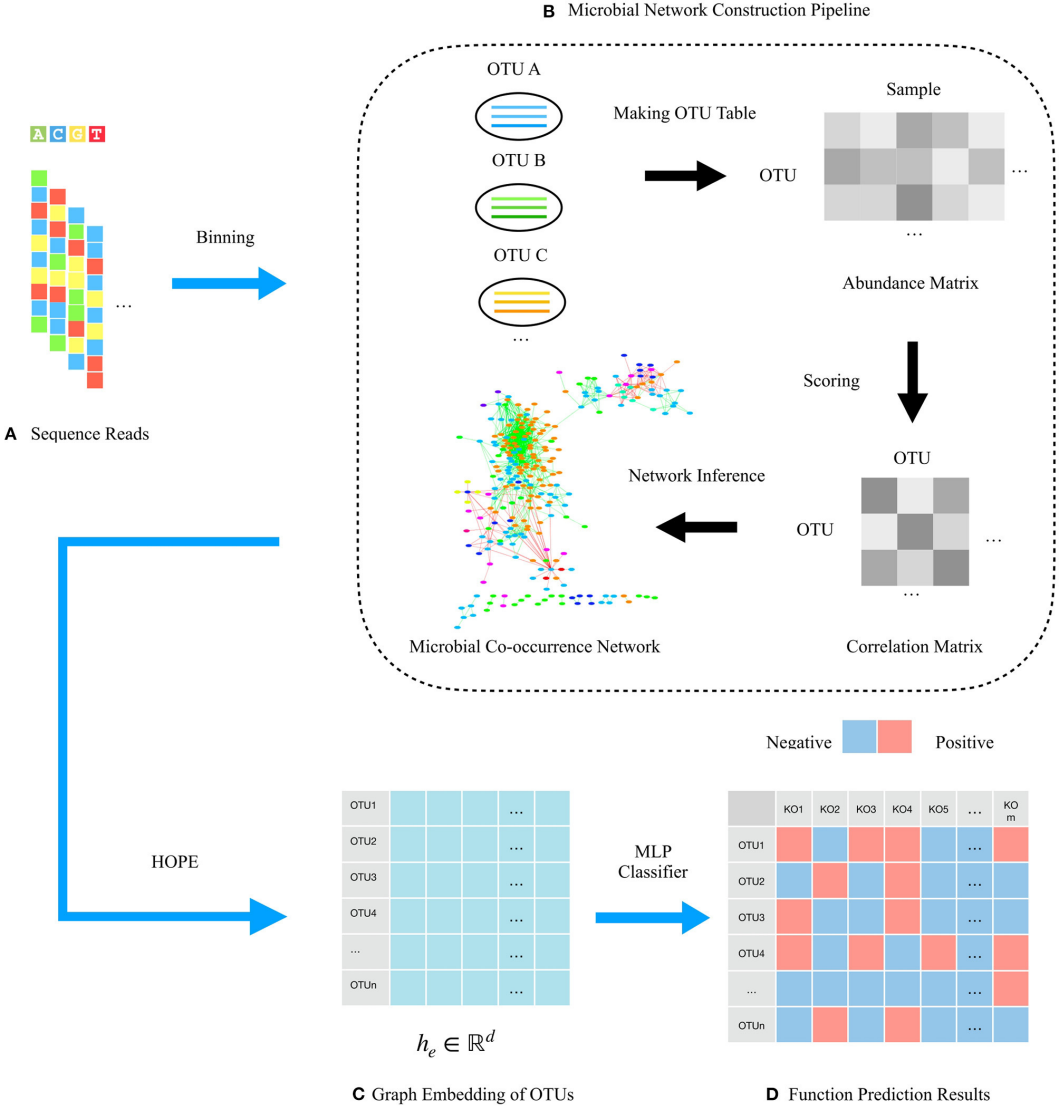
Schematic illustration of the framework for predicting microbial functions using HOPE. **(A)** 16s rRNA sequencing reads from a microbial community are adopted for network construction. **(B)** Pipeline for constructing microbial networks. OTUs are binned by clustering reads from the same source population. Then, the abundance matrix that describes the relative abundance of OTUs in every microbiota sample is calculated. Pairwise scores between OTUs are then computed gaining the correlation matrix, and OTU pairs with correlation score over the threshold are connected by an edge. Gray areas in the correlation matrix indicate similarity of OTUs. Finally, the whole microbial community is visualized as a network wherein nodes represent OTUs, and edges represent the correlation between them. **(C)** Embedding representations of each OTU via the HOPE algorithm. **(D)** Function prediction matrix of OTUs. Different colors indicate different KO functions.

HOPE consists of four steps to predict microbial functions, including data input, microbial co-occurrence network construction, graph embedding generation, and function prediction. The input are 16s rRNA sequence reads containing all microbial community information clustered to OTUs for building the microbial co-occurrence network based on the co-occurrence correlation relationships among OTUs. During the graph embedding step, HOPE learns the embedding vectors of the majority class and the minority class with multitask learning to mitigate class imbalance. The HOPE algorithm could distill the high-dimensional information about OTUs and their “neighbor OTUs” and embed the resultant data on topological structure into dense representative vectors. In this way, the original microbial network is converted to a compact embedding space, while a given node's features and the topological structure of its “neighborhood” are preserved. Finally, our approach uses the low-dimensional embedding vectors to identify microbial functions via an MLP classifier. The total pipeline of the framework is shown in Figure 1.

### Data Preprocessing

#### Construction of Microbial Co-occurrence Network

Before microbial function prediction can take place, the raw data coming from 16s rRNA sequencing data will be input to the framework, which may have millions of reads and cause numerous computations. The 16s rRNA sequences are grouped into OTU bins based on the sequence alignment similarity, which is a step that can reduce the number of OTUs for faster calculation. During the experiments, sequences are clustered to OTUs satisfying the following criteria via the UPARSE-OTU algorithm (Edgar, 2013). The sequences in the same cluster (OTU) should have more than 97% pairwise sequence alignment similarity, and the sequences in a different cluster (OTU) should have more than 3% pairwise sequence alignment dissimilarity. The OTU representative sequence is the most abundant contig in the OTU cluster and is selected to represent the cluster for the following processing. Then, the “co-occurrence” patterns, which were revealed as the co-occurrence interaction of two species or any taxonomically relevant units in habitats, are calculated via the OTU table and the correlation algorithm. The OTU table describes the abundances of OTUs in samples by the USEARCH algorithm (Edgar, 2010), and the correlation score is computed for each OTU pair by the SparCC algorithm (Friedman and Alm, 2012). OTUs with a higher correlation score than the threshold are considered proof of having a strong co-occurrence correlation, and these OTUs will be connected with an edge in the microbial network. The microbial co-occurrence network is constructed to preserve the interaction patterns where each node represents an OTU, and each edge represents a pairwise association between them and the pipeline as shown in Figure 1B.

The OTU representative sequences offer sequence signatures and potential information about their functions. *K*-mer means nucleotide sequences of length *k*. The *k*-mer frequency is the number of occurrences of *k*-mer within the whole sequence(s) normalized by the total number of occurrences in the vector for each data. The *k*-mers frequency is adopted as OTU features, whose statistical distribution of frequency reflects the sequence signatures. The short sequence representation, *k*-mers frequency, further reduces calculation and reflects the compositional distribution of DNA sequence(s). Previous studies have shown that *k*-tuple frequencies are similar across different regions of the same genome but differ between genomes (Karlin et al., 1997), which offers the theoretical basis to measure the dissimilarity between contigs. The length of k has a significant impact on the final results. When *k* ≥ 20 bp (long *k*-mer), *k*-mer reflects more detail and local biological information in the nucleotide sequences, but the high sparsity of the frequency vector lead by too long *k*-mer would lose the statistical power (Wang et al., 2014, 2018; Wang Y. et al., 2020). However, when *k* ≤ 10 bp (short *k*-mer), the frequency of *k*-mers reflects the global compositional distribution of the whole sequences (Ren et al., 2016). In our study, the representative sequence of each OTU is ~10^3^ bp; generally, *k* should be set from 4 to 10 (Wang et al., 2014). After testing on the different length of *k*-mer, the *k*-mer length of 7–10 has no much impact on performance. Therefore, we select *k* = 7 to reduce the running time of *k*-mer counting.

#### Function Labeling in Co-occurrence Network

As supervised learning, the labels of OTUs in the training set and the validation set need to be annotated. The multiclass classification means that there are more than two classes in the classification problem, and in our study, existence of multiple KEGG Orthology (KO) functions means multiple classes (Kanehisa and Goto, 2000). Multilabel means that a sample might belong to multiple classes, and in our study, there would be multiple KO functions for one OTU. Therefore, the function prediction task is formulated as a multiclass, multilabel classification problem. The label vectors containing the ground truth of OTU's functions utilize multihot encoding. This encoding approach could convert the useful information into a binary string with a single bit value of 1 or 0. If the OTU is annotated on the K00001 and K00003 function, then we will assign 1 to the first position and the third position in the binary string as a positive sample for this function. Every unique function category is represented as a binary value at a specific position in the labeled vector.

### Working Principle of HOPE

**Figure 2:**
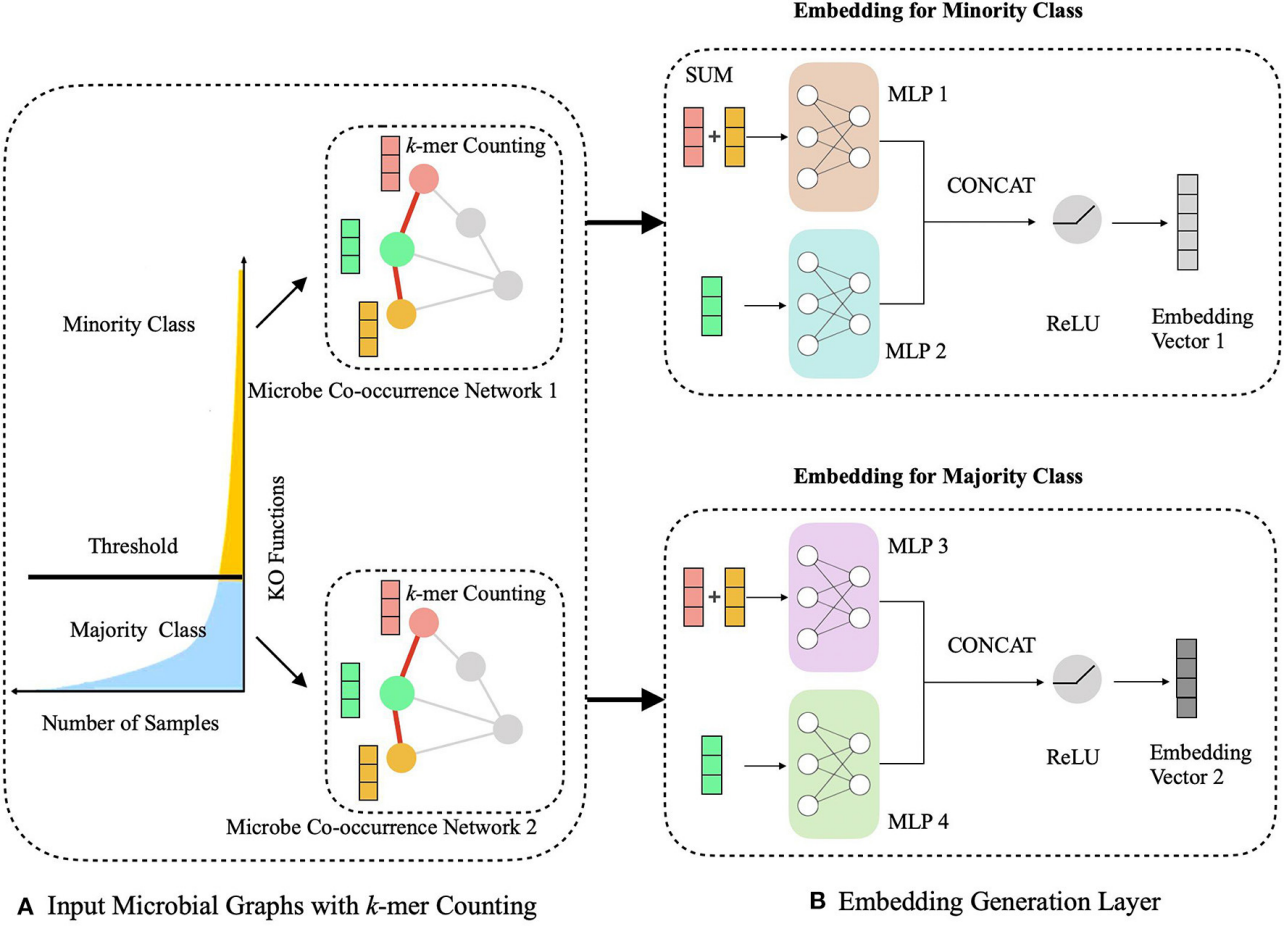
Schematic illustration of generating the embedding representations of microbial network with sequence k-mer counting. **(A)** We process the long-tailed distribution class with hierarchical multitask learning, which learns the majority class and the minority class independently with two microbial networks. **(B)** The embedding generation layer learns the embedding vector of OTUs via aggregating the sequence information from current nodes and their neighbors.

The HOPE algorithm includes two critical modules, a hierarchical multitask learning scheme and a graph embedding module (Figure 2). In the hierarchical multitask learning part, the HOPE model is trained on the majority class and the minority class, respectively, wherein the majority class means this class exists in more than half of OTUs, and the minority class only appears in less than half of OTUs. Then the graph embedding module learns embedding vectors of OTUs by propagating nodes' neighbor feature to the nodes along the edges and aggregating the topological structure of nodes' neighborhood with the *k*-mer representation of OTUs, along with the microbial co-occurrence network.

#### Hierarchical Multitask Learning Scheme

**Figure 3:**
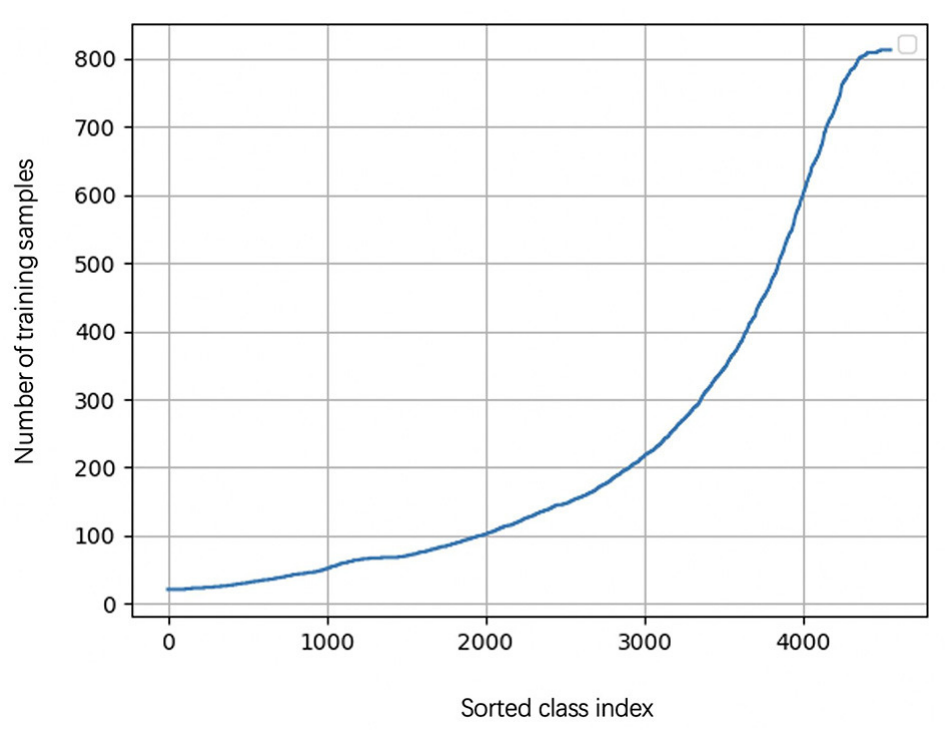
The KO function number of appearance cure. The cure is highly skewed because a few dominant function classes claim most of the samples, while most of the other function classes are represented by relatively a few samples.

During the learning of embedded representation of nodes, the highly skewed distribution of the functional class is observed (Figure 3), which will cause a class imbalanced problem. Long-tailed and skewed distributions among different functions cause the classifier to ignore the minority classes (Huang et al., 2016). The majority class will influence the classifier to be biased toward the majority class so that the minority class will be overwhelming, wherein the majority class means this class exists in more than half of OTUs, and the minority class only appears in less than half of OTUs. Traditional class rebalancing strategies, such as resampling and reweighting solutions, perform poorly on our tasks and slow down the training process. In fact, both majority and minority classes can be classified when trained independently. This motivated us to develop a hierarchical multitask training scheme designed to account for the poor prediction performance of minority classes (Figure 2A). The hierarchal multitask learning trains on the majority class and the minority class, respectively, so that the majority class in one task will not interfere the other task to learn the minority class and finally ameliorates the negative impact of the class imbalanced problem. The threshold of identifying a class belonging to a majority class or a minority class is a parameter that should be determined before model training, and the best threshold lets the model achieve the highest measurements on the validation set. Assume that the dataset is represented by space *V* × *Y*, where *V* indicates an OTU set with n OTU, and *Y* indicates the corresponding KO function set. The KO function set is then divided into the majority class *Y*_*ma*_ and the minority class *Y*_*mi*_ by the number of samples. The OTU set and the KO function set can be shown as

(1) $$\begin{array}{l} V=\left\{v_{1},v_{2},\ldots ,v_{n-1},v_{n}\right\} \end{array}$$

(2) $$\begin{array}{l} Y=Y_{ma}+Y_{mi} \end{array}$$

The goal is to learn two functions, *f*_1_, *f*_2_, that classify every input data point to the proper classes:

(3) $$\begin{array}{l} y_{mai}\approx f_{1}\left(v_{i}\right),i\in \left\{1,\ldots ,n\right\} \end{array}$$

(4) $$\begin{array}{l} y_{mii}\approx f_{2}\left(v_{i}\right),i\in \left\{1,\ldots ,n\right\} \end{array}$$

Two models are considered to learn the majority class and the minority class from the long-tailed dataset separately and simultaneously. The input data *v*_*i*_ are the same for all tasks, but the output values *y*_*i*_ are different for each task so that the novel method can mitigate the biased tendency of the classifier toward either the majority or minority class. The HOPE approach uses cross-entropy loss function as the feedback information to train to learn the embedding vector. A drop in the loss value means less bias between predicted values and observed targets:

(5) $$Loss_{ma}=-\sum_{i=1}^{n}\left(y_{mai}log(f_{1}(v_{i}))+(1-y_{mai})log(1-f_{1}(v_{i}))\right)$$

(6) $$Loss_{mi}=-\sum_{i=1}^{n}\left(y_{mii}log(f_{2}(v_{i}))+(1-y_{mii})log(1-f_{2}(v_{i}))\right)$$

#### Embedding of Microbial Co-occurrence Network

Based on the hierarchical learning framework, HOPE also computes the embedding vectors of microbial co-occurrence networks (Figure 2B). Our learning model for graph embedding builds upon the GraphSAGE (Hamilton et al., 2017) algorithm, which performs learnable aggregation to replace full-graph Laplacian and finds the embedding map for a large graph. Our algorithm integrates topological information for neighbors of each node with its own sequence information and conserves the useful graph data as completely as possible. Embedding vectors not only save node information but also save the graph's edge information. HOPE maps two nodes to close points in the embedding space if and only if their features are highly similar and their neighborhoods are topologically similar. These closed OTUs in the embedding space have a high probability of similar functions. Thus, the embedding vectors could be used intuitively for classification.

Graph embedding involves two key steps. First, randomly select the neighbor nodes of the target nodes and aggregate the features of these nodes with those of the target nodes via a SUM function. The microbial co-occurrence network has a feature set $h=\left\{h_{1},h_{2},\ldots ,h_{n}\right\},h_{i}\in {\mathbb{R}}^{f}$, where n denotes the number of nodes in the graph. We uniformly sampled *N* nodes to pick out a fixed-size set of neighbor nodes *V*_*N*_:

(7) $$\begin{array}{l} {\;V}_{N}=N\left(v\right) \end{array}$$

A sum aggregator function is used to combine these features of neighboring nodes, and we gain the aggregated representation of neighbor *h*_*N*_:

(8) $$\begin{array}{l} h_{N}=Aggregator\;(\left\{h_{i},\forall i\in V_{N}\right\}) \end{array}$$

The node's neighborhood embedding should be unique when no isomorphic neighborhoods exist. To aim this target, the aggregator function in the graph embedding algorithm has to be injective to achieve the upper bound method, the Weisfeiler–Lehman (WL) graph isomorphism test (Xu et al., 2019). Although the WL test has powerful capability in discriminating different graph structures, it does not know how to learn the intrinsic properties of nodes in a graph and generates unsuitable node features, which might be quite essential for function prediction task in testing. Thus, the WL test has poor generalization and would not be used in our study. The SUM aggregator that is used in this work is injective so that our method could be maximally powerful from a theoretical perspective and have well generalization. After aggregating features of the neighboring nodes, we then concatenate the target nodes feature, *h*_*T*_, with the aggregated neighbor feature, *h*_*N*_, and the concatenated vector is imported into the MLP layer with non-linear activation function σ:

(9) $$\begin{array}{l} h_{E}=\sigma \;(\left[W_{1}\cdot h_{T}\right]\;CONCAT\;[W_{2}\cdot h_{N}]) \end{array}$$

**Figure 4:**
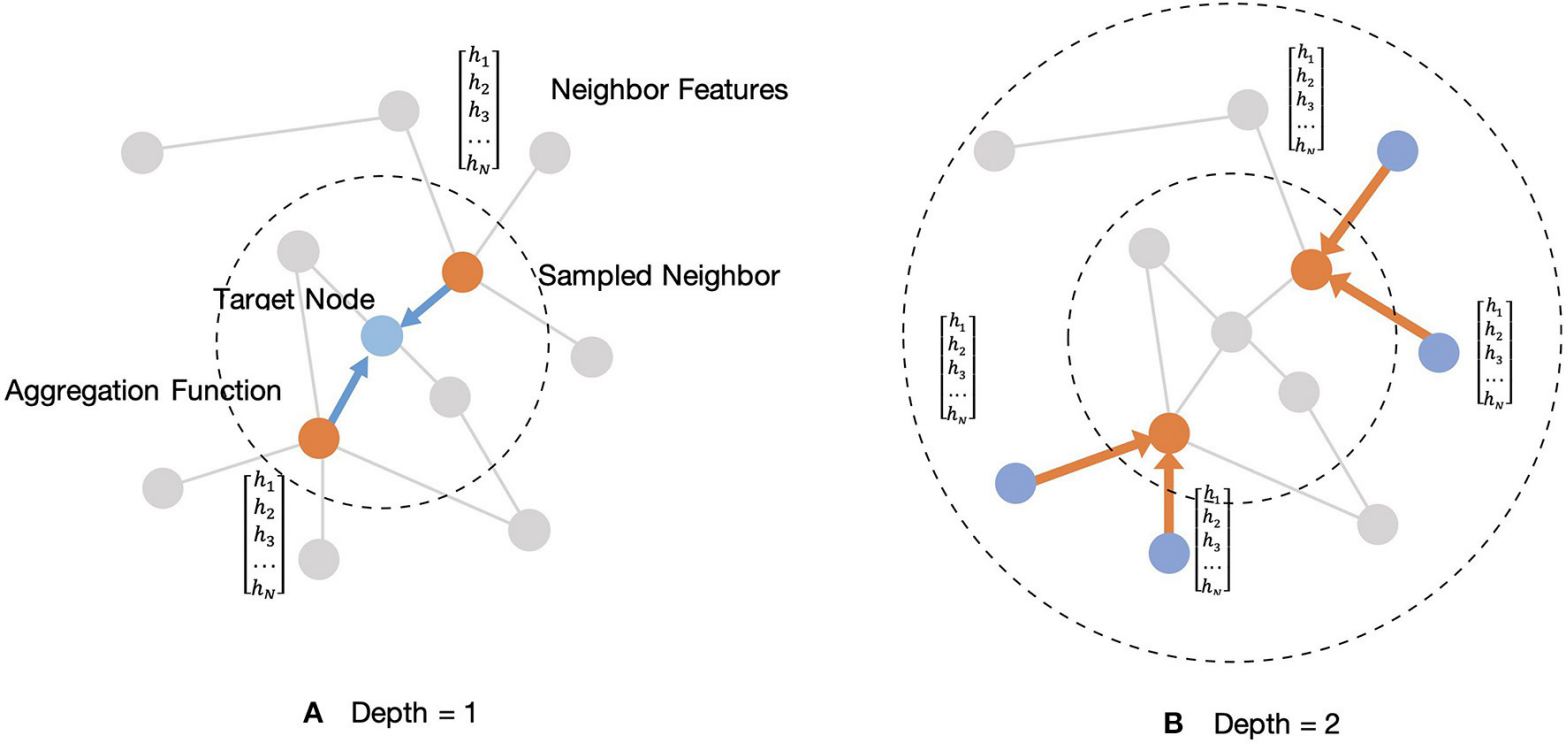
Illustration of sampled two-hop neighborhood and aggregation of features for these nodes. **(A)** Example of aggregating one-hop sampled neighborhood. **(B)** Example of aggregating two-hop sampled neighborhood.

*W*_*p*_, *p* ∈ {1, 2} are a set of weight matrices containing trainable weights that can be learned by back-propagation. This embedding generation process will be iterated in a loop as the searching depth deepens, K. For each iteration, target nodes will aggregate features from neighboring nodes to update the representation of a node, and the target node will gradually capture more and more information from further reaches of the nodes of the graph after two iterations of aggregation. After aggregating feature information from neighboring nodes in depth K, the layer outputs new embedding node features, as $h_{EK}=\left\{h_{e1},h_{e2},\ldots ,h_{en}\right\},h_{ei}\in {\mathbb{R}}^{d},d<F$. Then in the next iteration, the outputs feature *h*_*EK*_ from the previous depth would be considered as the neighboring features in depth K-1, *h*_*N*(*K*−1)_, and they will be aggregated with new target features, *h*_*T*(*K*−1)_, for updating. Thus, for the embedding representation vector, we get a target node feature after iterations. Figure 4 shows an example of an aggregating target node with its neighbors in two depths.

## Results

### Experimental Design

#### The Experimental Datasets

In this study, three 16s rRNA sequencing datasets from abalone gut, human gut from early pregnancy, and *P. monodon* gut are tested in the experiments. The three datasets are available in NCBI with accession IDs ERP017548, SRP266217, and SRP261546. We constructed the microbial networks of the datasets by co-occurrence correlation, and the detail of these networks is shown in Table 1.

**Table 1:** Summary of the datasets used in our experiments.

	**Abalone gut**	**Human gut**	***Penaeus monodon* gut**
Nodes	15,796	3,254	42,84
Average edges	41.4	6.08	18.05
Classes	4,075	4,289	5,144
Training nodes	11,058	2,278	2,999
Validation nodes	3,159	649	855
Test Nodes	1,579	327	430

#### Experimental Strategies

Before making a comparison of specific methods, we first take steps to confirm our key hypothesis, i.e., that the co-occurrence relationship among microbes, together with neighborhood topological structures in the microbe network, provides a fully functional context for function prediction. After that, to further evaluate the performance of our method, we design two experimental strategies: function prediction within a microbial community and function prediction across the microbial community. In the first strategy, both the training set and the test set come from the same microbial community to check the normal prediction ability of our method. The second strategy trains the model on one type of microbial community and then tests that model on a different, but related, microbial community, aiming to test the generalization ability of HOPE. Thus, HOPE must learn the universal knowledge on the training set and the validation set to make sound prediction results on the test set.

Function prediction for each OTU is modeled as a multiclass, multilabel supervised classification problem. In our study, the experimental dataset is divided into three distinct parts, including the training set, the validation set, and the testing set. We randomly split 20% of all OTUs into an independent testing set and designed an eight-fold cross-validation on the remaining 80% of all OTUs. The cross-validation is applied to learn the appropriate parameters in the weight matrices and select the appropriate hyperparameters. The goal is to develop the best model on both training and validation sets to achieve the highest prediction performance on the testing set. All methods mentioned above use rectified linear units (ReLUs) as the non-linearity functions to evaluate all datasets.

#### Hyperparameters of the Training Process

In training, we use the cross-entropy loss function for multiclass, multilabel classification together with the Adam optimizer (Kingma and Ba, 2015). The cross-entropy loss function treats each class independently and measures the difference between the ground truth label and predicted labels. The ground truth label of each class is 0 or 1, and the predicted result of each class is the probability between 0 and 1. When the predicted probability is far from the ground truth label, the loss value will be large.

We set *K* = 2 as the neighborhood region and sample sizes S1 = 25 and S2 = 10 at each hop of a neighborhood leading to the best performance during the graph embedding step. Adam and L2 regularization are adopted for model optimization with the size of mini batch at 128 and a learning rate of 0.01. To avoid overfitting, dropout is set as = 0.4. All experiments use ReLUs as activation functions. The experiments are run on a single machine with 4 NVIDIA GeForce GTX1080 TI with CUDA Version 10.2, Intel(R) Xeon(R) CPU (E5-2620 v4 @ 2.10 GHz), and 128 Gb of RAM.

### Hypothesis Verification

As noted above, this work is driven by the hypothesis that microbes with strong correlations, or strong neighborhood topology profiles, have similar, or highly correlated, functions. Therefore, we designed the following experiments to confirm that the topological structure of a neighbor node is predictive, or not, by comparing the similarity of KOs between OTU nodes with similar and different neighbors.

#### Verify Whether Two Adjacent Neighbors Share Highly Correlated Functions

**Figure 5:**
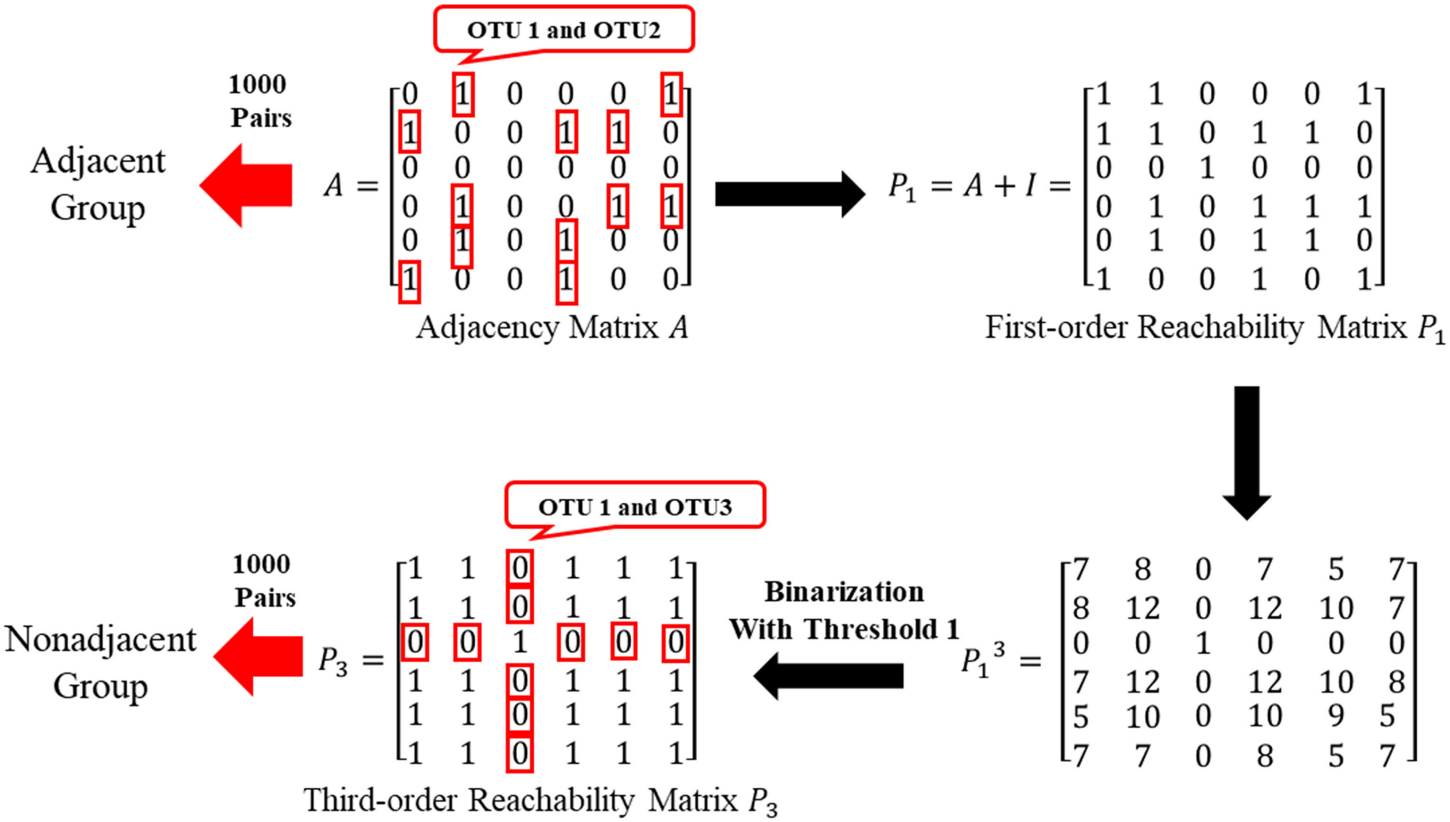
Sketch of extracting “Adjacent Group” and “Non-adjacent Group”.

**Figure 6:**
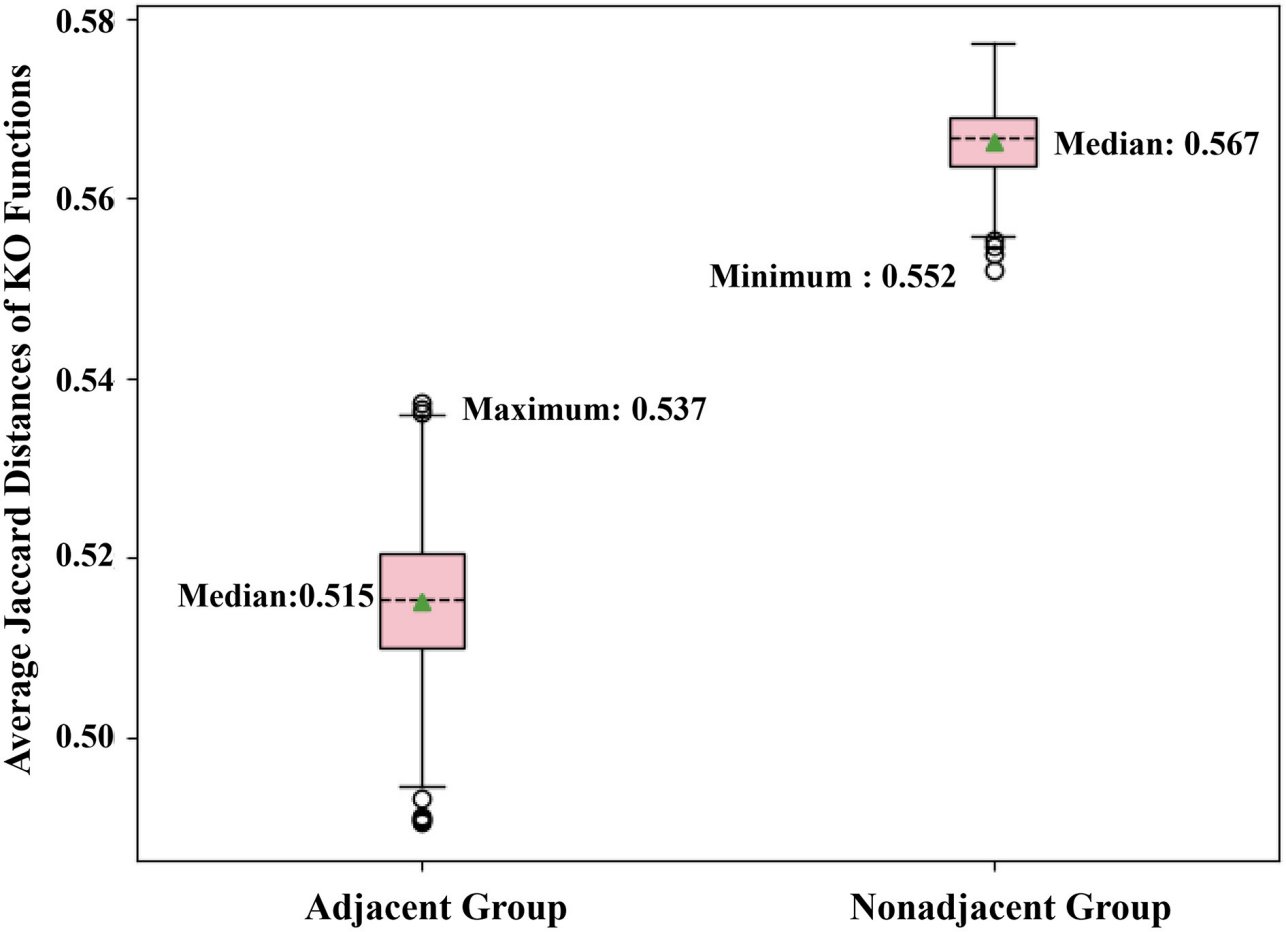
Box plot comparing KO similarities 1,000 times with each test extracting 1,000 pairs of nodes for two groups and calculating the average Jaccard distance.

By the adjacent matrix of the microbial network, 1000 pairs of adjacent OTU nodes are randomly extracted as the “Adjacent Group.” Meanwhile, a three-step reachability matrix of OTUs is computed from the adjacency matrix. The two OTU nodes that cannot reach each other within three steps are extracted as the “Non-adjacent Group,” which ensures that the node pairs are sufficiently far from each other. The extraction process of the two groups is shown in Figure 5. OTU functions are represented by a row of KO function vectors using 0/1 to indicate whether the OTU possesses the KO function or not. The distance between function vectors of two OTUs is calculated by the Jaccard distance. The function distances between the “Adjacent Group” and the “Non-adjacent Group” are calculated and averaged, respectively, and the tests are repeated 1,000 times. Figure 6 shows the average function distances from the “Adjacent Group” and the “Non-adjacent Group” over the course of 1,000 respective tests. The median of average Jaccard distances of the “Adjacent Group” is 0.515, which is significantly lower than that of the “Non-adjacent Group.” Even the maximum average distance from the “Adjacent Group” is smaller than the minimum average distance from the “Non-adjacent Group,” which suggests that the adjacent relationships of OTU nodes contain the information required to predict KO functions.

#### Verify Whether Two Nodes Sharing Similar Neighbors Would Have Highly Correlated Functions

**Figure 7:**
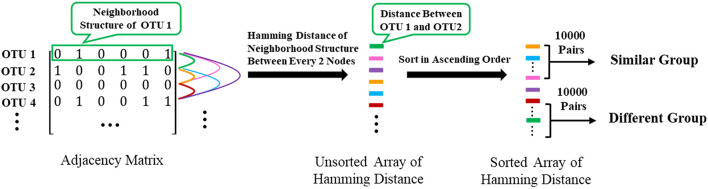
The extraction of “Similar Group” and “Different Group”.

**Figure 8:**
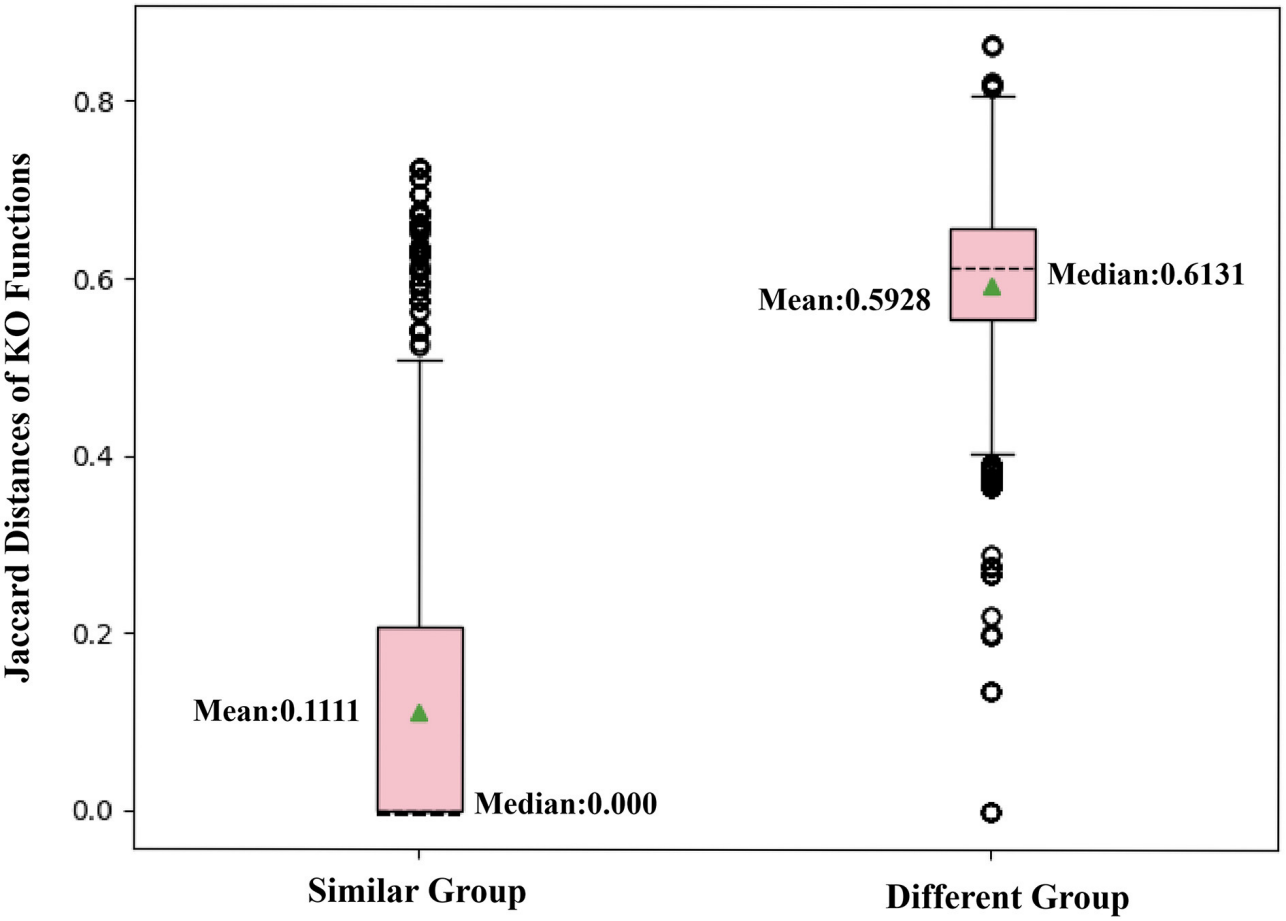
Box plot comparing KO similarities by calculating Jaccard distances with each group, including 10,000 pairs of OTUs with similar or different neighbors.

To further confirm that two nodes sharing similar neighbors have highly correlated functions, we use the corresponding row of the adjacent matrix to present the neighbor structure of each OTU. Hamming distance between every two rows of the adjacent matrix is adopted to evaluate neighborhood similarity between two corresponding OTUs. The smaller the Hamming distance between the two rows is, the more similar the neighborhood of the two nodes is. As shown in Figure 7, we selected the 10,000 pairs of nodes with the smallest Hamming distance in the neighborhoods as the “Similar Group” and the 10,000 pairs with the largest Hamming distance in the neighborhoods as the “Different Group.” Therefore, OTU pairs in the “Similar Group” share similar neighbors, and the other pairs in the “Different Group” do not. Function similarity is also measured by the Jaccard distance between KO function vectors. Function distances for the “Similar Group” and the “Different Group” are calculated and plotted as boxplots, as shown in Figure 8. It is clear that KO functions are closer to each other for OTUs sharing common neighbors. For OTUs with highly different neighbors, KO functions are farther apart. The mean of Jaccard distances of the “Similar Group” is 0.1111, which is much smaller than that of the “Different Group.” Based on the two verification experiments, we infer that the OTUs that are adjacent to, or share, common neighbors would have highly similar KO functions. Therefore, learning the topological structure of the microbial co-occurrence network would provide clear and beneficial information for function predictions.

### Evaluations and Comparisons of Experimental Results

HOPE, its two variants, HOPE-seq and HOPE-one, and GraphSAGE are applied to three datasets to evaluate by comparison the performance of HOPE. Recall that HOPE features hierarchical multitask learning to solve the highly skewed class distribution problem, and it incorporates information of both microbe sequences and microbe interactions in a co-occurrence network. Therefore, the variant HOPE-seq only uses the microorganism sequence representations as input but utilizes hierarchical multitask learning to train the classifier on majority and minority classes. HOPE-one ignores class imbalance problems but integrates the vector representation of sequences with microbial network information as an input feature. Both of the variant methods use the same hyperparameters and training strategies as parent HOPE. GraphSAGE (Hamilton et al., 2017) is a well-known and widely used graph embedding algorithm that provides an inductive framework to generate embeddings by sampling and aggregating features from a node's local neighborhood. The aggregation function can have various forms, and the authors suggest three aggregator functions: a mean aggregator, an LSTM aggregator, and a pooling aggregator (shown as GS-Mean, GS-LSTM, and GS-Pooling, respectively). The mean aggregator simply takes the elementwise mean of the node's features. The LSTM aggregator is built on a standard LSTM architecture (Hochreiter and Schmidhuber, 1997) to aggregate the nodes' neighbors, which are listed to a random permutation, to embedding representations. The detailed description of the LSTM aggregator can be found in the study of GraphSAGE (Hamilton et al., 2017). In the pooling aggregator, an elementwise max-pooling operation is applied to aggregate information across the node's neighbors.

In our experiments, we use four different measurements, including micro-averaged F1 score, macro-averaged F1 score, accuracy, and ROC-AUC score, to judge the comparison results. Micro-averaged F1 score and macro-averaged F1 score are both F1 scores, but they differ in the averaging method.

The micro-F1 score will aggregate the true positive (TP), false positive (FP), true negative (TN), and false negative (FN) of all classes to compute the average F1-score. Assuming n classes, the TP-value, FP-value, and TF value of the ith class are represented as *TP*_*i*_, *FP*_*i*_, and *FN*_*i*_, respectively:

(10) $$\begin{array}{l} {precision}_{mi}=\frac{\overset{n}{\underset{i=1}{\sum }}{TP}_{i}}{\overset{n}{\underset{i=1}{\sum }}{TP}_{i}+\overset{n}{\underset{i=1}{\sum }}{FP}_{i}} \end{array}$$

(11) $$\begin{array}{l} {recall}_{mi}=\frac{\overset{n}{\underset{i=1}{\sum }}{TP}_{i}}{\overset{n}{\underset{i=1}{\sum }}{TP}_{i}+\overset{n}{\underset{i=1}{\sum }}{FN}_{i}} \end{array}$$

(12) $$\begin{array}{l} micro-F1=2\frac{{recall}_{mi}\times {precision}_{mi}}{{recall}_{mi}+{precision}_{mi}} \end{array}$$

On the other hand, the macro-F1 score will compute the F1-score independently for each class and then take the average as

(13) $$\begin{array}{l} {precision}_{i}=\frac{{TP}_{i}}{{TP}_{i}+{FP}_{i}} \end{array}$$

(14) $$precision_{ma}=\frac{\sum_{i=1}^{n}precision_{i}}{n}$$

(15) $$\begin{array}{l} {recall}_{i}=\frac{{TP}_{i}}{{TP}_{i}+{FN}_{i}} \end{array}$$

(16) $$recall_{ma}=\frac{\sum_{i=1}^{n}recall_{i}}{n}$$

(17) $$\begin{array}{l} macro-F1=2\frac{{recall}_{ma}\times {precision}_{ma}}{{recall}_{ma}+{precision}_{ma}} \end{array}$$

Accuracy is the ratio of correct predictions to total input samples. The ROC-AUC score is defined as the area under the ROC curve. It provides an aggregate measure of performance across all possible classification thresholds. The ROC-AUC score varies between 0 and 1, and the closer it is to 1, the better the performance of the classifier.

#### Functional Prediction Within the Same Microbial Community

In this part, we use the training, validation, and testing data from the same microbial community and calculate various measurements for every experiment (see Table 2). HOPE is compared against its variants and GraphSAGE. According to the results, HOPE nearly outperforms all baselines on various measurements, especially the micro-F1 score and macro-F1 score. However, the performance of HOPE is comparable to its two variants in terms of accuracy and ROC-AUC. For example, HOPE outperforms HOPE-one by 9.5% in the micro-F1 score on the Abalone Gut Microbiota dataset and 15.6% in the macro-F1 score on the *P. monodon* intestine dataset. In some parameters, the performance of HOPE-one is better than that of HOPE-seq, like accuracy and ROC-AUC, but HOPE-seq can improve upon HOPE-one by a margin of 5.7% in the micro-F1 score on the Abalone Gut Microbiota. Since HOPE integrates sequence information with microbial network information via graph embedding, thus combining the advantages of its two variants, it nearly achieves the highest performance. Table 2 also shows the performance results of HOPE compared to the variants of GraphSAGE on the benchmark datasets. HOPE nearly achieves the highest score in all measurements and outperforms two baselines by a significant margin. According to Table 2, we find that HOPE-one achieves better results on accuracy and ROC-AUC than HOPE on three datasets because HOPE sacrifices some performance on the majority class to learn the minority class well.

**Table 2:** The performance of HOPE and its variants and GraphSAGE within the same microbial community for training and testing.

**Method**	**Abalone gut microbiota**				**Human feces**				***Penaeus monodon*** **intestine**			
	Mi- F1*	Ma-F1*	**Accuracy**	**ROC-AUC**	**Mi-F1**	**Ma-F1**	**Accuracy**	**ROC-AUC**	**Mi-F1**	**Ma-F1**	**Accuracy**	**ROC-AUC**
HOPE-seq	0.786	0.500	0.887	0.840	0.742	0.236	0.861	0.807	0.907	0.701	0.941	0.923
HOPE-one	0.729	0.544	**0.921**	**0.881**	0.742	0.199	0.883	0.816	0.941	0.675	0.963	**0.955**
GS-mean	0.727	0.433	0.861	0.822	0.672	0.217	0.843	0.780	0.872	0.515	0.918	0.908
GS-LSTM	0.677	0.290	0.843	0.777	0.711	0.205	0.867	0.800	0.713	0.263	0.829	0.787
GS-pooling	0.735	0.387	0.879	0.806	0.747	0.183	**0.888**	**0.816**	0.738	0.263	0.845	0.804
HOPE	**0.824**	**0.592**	0.908	0.869	**0.758**	**0.309**	0.870	0.811	**0.941**	**0.831**	**0.963**	0.949

* *Mi-F1 and Ma-F1 represent micro-F1 score and macro-F1, respectively. The bold values mean the best performance of each column of index*.

#### Functional Prediction Across Different Microbial Communities

We further consider generalizing across different microbial communities, which requires our model to learn the context of common functions from one microbe to infer the functions of other organisms. Some researchers may want to know novel microbial functions but have only information about related microbial functions. In this case, the generalization ability of the algorithm is very important. Therefore, in this part, we design experiments with the different test sets to evaluate the generalization ability of HOPE.

We first set the training and validation data from the abalone gut microbiota and use human feces and shrimp intestine microbiota to construct microbial networks as a test set. In these scenarios, we evaluate the performance of our model when the training data are different from the data used in the test set. Table 3 summarizes the performance of HOPE with different test sets. Compared to baselines, experiments utilizing a test set different from the training set achieve lower scores but within an acceptable range. We train the model on the abalone gut microbiota dataset and test the model on datasets from human feces and shrimp intestine microbiota. Although using training sets from a different source, results show that HOPE achieves nearly 90% performance of experiments when the training set and test set data belong to the same species. HOPE achieves high performance in generalization, which means that our approach can learn the fundamental knowledge from known microbial functions and infer the functions of unseen microorganisms.

**Table 3:** Evaluation of generalization performance of HOPE across different microbial communities.

**Training set organism**	**Test set organism**	**Mi-F1**	**Ma-F1**	**Accuracy**	**ROC-AUC**
Human feces	Human feces	0.758	0.309	0.842	0.811
Abalone gut	Human feces	0.728	0.217	0.867	0.819
*Penaeus monodon* intestine	*Penaeus monodon* intestine	0.941	0.831	0.963	0.949
Abalone gut	*Penaeus monodon* intestine	0.837	0.529	0.877	0.866

*The first and second columns list the microbial communities used in the training set and the testing set, respectively. The first and third rows list the baseline of performance when training and test sets are from the same microbial community*.

#### Discussion for Class Imbalance Problem

**Figure 9:**
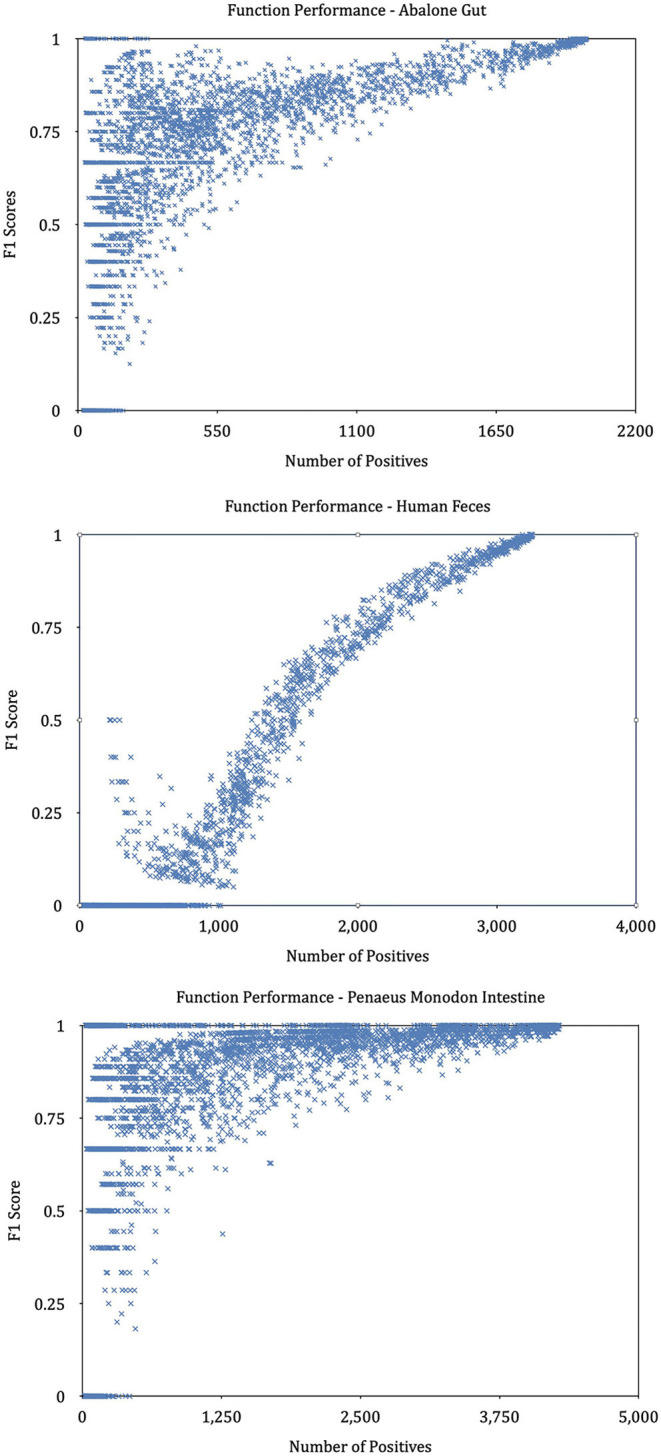
Performance of KOs with different annotated samples. The graphs plot the predictive performance of each KO in our method as a function of the number of training samples.

We find that KO functions with a large number of annotation samples generally outperform KO functions with a few annotations. Further experiments explore the relationship between the number of training samples and the variance in predictive performance and plot the result in Figure 9. It can be seen that the predictive performance is strongly correlated with the number of instances in the training set. We build linear regressions for the measurement scores and the number of samples for every KO function, and the coefficients of the explanatory variable in all regressions are significantly greater than zero (*P* = 0.0000). The statistical results prove that KO functions with rich training sample annotations perform better than KO functions represented by only a few samples.

In all experiments mentioned above, we observe that some specific KO functions are easily classified to wrong places, causing low scores across the measurements evaluated. Even though some specific KO functions have been learned by a large amount of training samples, like K00096, K02080, and K10014, their F1 scores are nearly zero. Owing to the hierarchical nature of KOs, these bad KOs are defined as low-level, or rare, existing functions. In the future, additional weights based on the general level of KO should be assigned to each class to achieve better performance.

## Conclusion and Discussion

In this paper, a pipeline for the HOPE method is proposed for the analysis of microbial functions. The method leverages hierarchical multitask learning and graph embedding to extract features from sequence compositional signatures and topological patterns in non-linear microbial interaction networks. The hierarchical multitask learning module is to cope with class imbalanced datasets and achieve significant performance gains on predicting functions that appear in a few training samples. Using the graph embedding model, HOPE integrated the sequence compositional signatures and co-occurrence relationship among OTUs in microbial communities with the *k*-mer frequency feature in each node and topological patterns in microbial networks. Therefore, HOPE outperforms baselines on almost all indexes in all experiments. In detail, the percentage of macro-F1 scores reached from our classifier has an increased score of at least seven percentage points compared to the other methods. Experiment results also showed that HOPE has satisfactory generalization ability when it predicts functions across different microbial communities. Because the graph embedding of microbial co-occurrence networks conserves the interactions and similarities among OTUs, which are useful for inferring unknown functions, HOPE demonstrates significant generalization ability. Several potential improvements are possible. In the future, during the construction of the microbial network, the threshold of defining an edge between two OTUs could change to a learnable value. The training of HOPE is more time-consuming than the previous algorithms because the extra MLP layer for function prediction requires the optimization of much more parameters.

Although the primary purpose of HOPE is the prediction of microbial functions on the microbial co-occurrence network, the framework can be used on other related scenarios, such as the prediction of gene function based on the gene co-expression network.

## Data Availability Statement

The original contributions presented in the study are included in the article/supplementary material, further inquiries can be directed to the corresponding author/s.

## Author Contributions

YW, YH, XZ, and QZ planned the project. YW and YH designed the model and experiments. YH, XZ, and QZ performed the experiments. YW, YH, and QZ analyzed the data. YH and WH contributed the materials and analysis tools. YW, YH, and QZ wrote the main manuscript. All authors read and approved the final manuscript.

## Conflict of Interest

The authors declare that the research was conducted in the absence of any commercial or financial relationships that could be construed as a potential conflict of interest.
